# A Study of Dry Mouth and Gastrointestinal Disorders in Patients Taking Antidepressant

**DOI:** 10.1192/j.eurpsy.2024.1033

**Published:** 2024-08-27

**Authors:** S.-Y. Lee, H.-J. Lee

**Affiliations:** ^1^Psychiatry, Wonkwang University Hospital; ^2^Public Health, Wonkwang University Graduate School, Iksan, Korea, Republic Of

## Abstract

**Introduction:**

Dry mouth is a subjective symptom of the feeling of dehydration inside of the mouth and is closely linked to reduced salivary secretion. The occurrence of dry mouth and GI disorders due to antidepressants greatly affects the course of the mental disorder and medication compliance, but it has barely ever been studied.

**Objectives:**

The purpose of this study was to identify the characteristics of dry mouth and gastrointestinal (GI) disorders in antidepressant patients.

**Methods:**

The study included 103 antidepressant-taking patients. Antidepressants were classified according to their mode of action. The GI disorders were investigated using the medical records of the patients. The Patient Health Questionnaire-15 and a questionnaire for assessing dry mouth symptoms were used in this study. The questionnaire for the evaluation of dry mouth symptoms, a visual analog scale (VAS)–based instrument, developed and evaluated for reliability by Lee et al. was used to assess dry mouth. In the questionnaire, 6 VAS items were assessed for the extent of dry mouth (0-100 points) : 1) dry mouth at night or when waking up in the morning, 2) dry mouth during the day, 3) dry mouth when eating, 4) difficulty in swallowing, 5) subjective evaluation of the volume of saliva in the mouth, and 6) overall discomfort in daily life. Additionally, four items examined behaviors due to dry mouth (1-5points) : 1) frequency of waking up from sleep due to dry mouth, 2) frequency of preparing drinking water before going to bed, 3) frequency of drinking water when eating solid foods, and 4) frequency of eating hard candies or chewing gums to help dry mouth.

**Results:**

The score for “overall discomfort due to dry mouth in daily life” (31.72±33.82), “dry mouth at night or in the morning” (47.86±35.87), and “dry mouth during the day” (39.83±31.67) were slightly higher than “discomfort in chewing or swallowing foods”. According to somatization severity, the mean values were 116.36±113.34 in the mild, 213.18±136.98 in the moderate, and 277.59±201.44 in the severe, the between-group difference was significant (F=10.294, p<0.001). According to the class of antidepressants, the mean score was 180.00±147.5 for vortioxetine, 194.25±169.33 for selective serotonin reuptake inhibitors (SSRIs), 223.61±156.70 for serotonin and norepinephrine reuptake inhibitors (SNRIs), 75.00±57.00 for norepinephrine dopamine reuptake inhibitors (NDRIs), 201.67±174.66 for Nassau, and 116.67±132.03 for agomelatine. A total of 67 (65.0%) patients had at least one GI disorder.

**Conclusions:**

The study findings are expected to help increase medication compliance in antidepressant patients by better controlling the side effects experienced by the patients.

**Disclosure of Interest:**

None Declared

